# Analysis of Intact Glycosidic Aroma Precursors in Grapes by High-Performance Liquid Chromatography with a Diode Array Detector

**DOI:** 10.3390/foods10010191

**Published:** 2021-01-19

**Authors:** Cristina Cebrián-Tarancón, José Oliva, Miguel Ángel Cámara, Gonzalo L. Alonso, M. Rosario Salinas

**Affiliations:** 1 Cátedra de Química Agrícola, E.T.S.I. Agrónomos y Montes, Departamento de Ciencia y Tecnología Agroforestal y Genética, Universidad de Castilla-La Mancha, Avda. de España s/n, 02071 Albacete, Spain; cristina.ctarancon@uclm.es (C.C.-T.); gonzalo.alonso@uclm.es (G.L.A.); 2 Departamento de Química Agrícola, Geología y Edafología, Facultad de Química, Universidad de Murcia, Campus de Espinardo s/n, 30100 Murcia, Spain; josoliva@um.es (J.O.); mcamara@um.es (M.Á.C.)

**Keywords:** aroma precursors, grapes, HPLC-DAD, HPLC-qTOF-MS, intact glycosides

## Abstract

Nowadays, the techniques for the analysis of glycosidic precursors in grapes involve changes in the glycoside structure or it is necessary the use of very expensive analytical techniques. In this study, we describe for the first time an approach to analyse intact glycosidic aroma precursors in grapes by high-performance liquid chromatography with a diode array detector (HPLC-DAD), a simple and cheap analytical technique that could be used in wineries. Briefly, the skin of Muscat of Alexandria grapes was extracted using a microwave and purified using solid-phase extraction combining Oasis MCX and LiChrolut EN cartridges. In total, 20 compounds were selected by HPLC-DAD at 195 nm and taking as a reference the spectrum of phenyl β-D-glucopyranoside, whose DAD spectrum showed a first shoulder from 190 to 230 nm and a second around 200–360 nm. After that, these glycosidic compounds were identified by High-performance liquid chromatography–quadrupole time-of-flight mass spectrometry (HPLC-qTOF-MS). Disaccharides hexose pentose were the most abundant group observed with respect to the sugars and monoterpendiols the main aglycones found.

## 1. Introduction

Aroma glycosides are compounds produced during the secondary metabolism in plants formed by a sugar moiety linked to a volatile aglycone by a β-glycosidic bond. The main sugar is β-D-glucose however, another sugar molecule may be added to form higher glycosides such as disaccharide, which have been indicated as the most abundant in grapes [1].

In glycosylated aroma precursors of grapes, the aglycone moiety is responsible for the potential aromatic character of the molecule since, although these compounds originally have no odour, the volatile aglycones are released during winemaking contributing to the wine aroma profile [2,3]. The aglycone can belong to different chemical families, such as terpenes, phenols or norisoprenoids [1] and even though their nature depends mainly on the grapes’ variety, it may also be influenced by other factors, such as soil or climatic conditions or viticultural practices. In fact, several studies have proven that it is possible to modify the glycosidic aroma profile of grapes by treating the leaves with oak or guaiacol extracts [4,5,6].

Such an important role of glycosylated aroma precursors has stimulated the development of analytical methods for their quantification [7]. Typically, such precursors are analysed by isolating their glycosides, followed by the release of aglycones via enzymatic hydrolysis [8,9] or acidic hydrolysis [10,11,12], and finally the analysis of volatile aglycones by gas chromatography-mass spectrometry (GC-MS). However, in both cases, the molecular structure of glycosylated compounds is modified during the analysis process, a partial breaks occur in the glycosides, generating fragments and causing major structural changes in the aglycones, which do not reflect the original glycoside chemistry of grapes [1,2].

In a different line, Serrano de la Hoz (2014) [13] developed a fast method for analysing the potential aroma of grapes (IPAv) by measuring the amount of glycosidic glucose released at equimolecular proportions of volatile aglycones by acid hydrolysis. Although the simplicity of this method makes it useful in viticulture and wine production, it provides an estimate of the total content of glycosidic aroma precursors, but not a concentration.

The firsts identifications of aroma precursors by high-performance liquid chromatography–mass spectrometry (HPLC-MS/MS) was described by Nasi et al. 2008 [14] and Schievano et al. 2013 [15] and later several researchers have been proposed different strategies for the direct quantification of aglycones using ultra-high-performance liquid chromatography–quadrupole time-of-flight mass spectrometry (UHPLC-QTOF-MS/MS) [16,17,18]. However, due to the complexity of these techniques and the high cost of the equipment needed, it is difficult to employ them in viticulture or wineries. For its part, the high-performance liquid chromatography with a diode array detector (HPLC-DAD) has been used for the quantification of glycosylated compounds in other crops, as for example steviol glycosides, which are glycoconjugated terpenols whose structures are very similar than glycoside terpene aroma precursors [19,20]. In these articles it is reported the maximum absorption wavelengths of these compounds in 210 nm, probably associated to the aglycone. But, to increase the sensitivity, measurements could be done at a lower wavelength (e.g., 195 nm), where sugars have their maximum wavelengths of absorption.

It could be a simple and cheap analytical technique that allows wineries to determine intact glycosidic aroma precursors but, to our knowledge, no previous studies on the use of this technique in grapes have been performed. Therefore, this work addresses the possibility of analyzing intact glycosidic grape aroma precursors by HPLC-DAD.

## 2. Materials and Methods

### 2.1. Grape Material

Fifty kilograms of Muscat of Alexandria grapes were collected in a representative way of the plot from a vineyard of O.D. La Mancha (Castilla-La Mancha, Spain) during the 2019 harvest under proper sanitary conditions and at the optimal stage of maturity. All grapes were immediately frozen at −20 ± 2 °C until extraction.

### 2.2. Extract Preparation

Grapes frozen were skinned, the pulp and seeds were removed, and the skins were freeze-dried (LyoAlfa 6–50; Telstar, Terrassa, Spain). Then, 20 g of homogenised freeze-dried skin from 0.5 kg of frozen grapes was ground to a fine powder and moisturised with 70 mL of water for 2 h at room temperature (20 ± 3 °C). Then, extraction was performed using a NEOS microwave device (Milestone, Sorisole, Italy) at 70 °C (600 W) for 1 min [21]. The mixture was then centrifuged at 4000 rpm (3000× *g*) for 10 min, and the total supernatant volume was taken and divided into two similar fractions of approximately 25 mL each, which is the adequate volume for each cartridge.

### 2.3. Isolation of Intact Glycosidic Aroma Precursors

**Figure 1:**
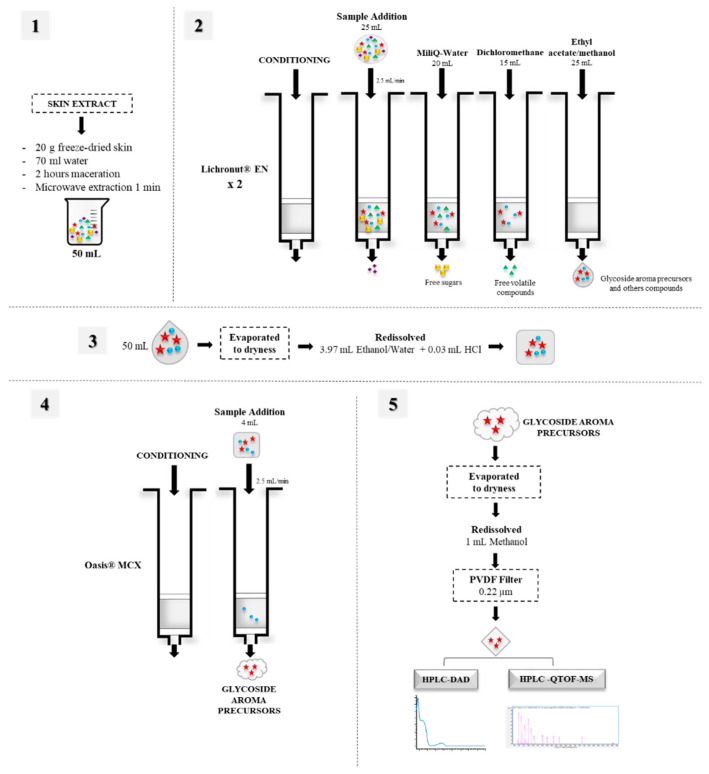
Extraction and isolation procedure of glycosidic aroma precursors.

Glycosidic aroma precursors were extracted and purified using solid-phase extraction (SPE) according to the method described by Hernández-Orte et al. (2015) [22], with minor modifications, as seen in Figure 1.

As a first step, each fraction (25 mL) was passed through a LiChrolut EN cartridge (40–120 µm, 500 mg; Millipore Corp., Bedford, MA, USA), followed by the use of an Oasis MCX SPE cartridge (60 µm, 500 mg; Waters Corp., Milford, MA, USA). The LiChrolut EN cartridge was conditioned with 16 mL of dichloromethane, 16 mL of methanol and 32 mL of Milli-Q water, and approximately 25 mL of skin extract was passed through the cartridge at a flow rate of 2.5 mL/min. Then, the cartridges were washed with 20 mL of water to remove free sugars, followed by 15 mL of dichloromethane to remove the free volatile fraction. The glycosidic aroma precursor fraction was then recovered using 25 mL of ethyl acetate/methanol (90:10, *v*/*v*). The two eluates were mixed and the total volume (50 mL) was evaporated to dryness using a rotary vacuum evaporator (LABOROTA 4000eco; Heidolph Instruments, Schwabach, Germany). Then, the dryness mixture was re-dissolved in 3.97 mL of ethanol/water (50:50, *v*/*v*) together with 30 µL of HCl (pH 1). To remove polyphenols, all solutions (4 mL) were passed through an Oasis MCX cartridge previously conditioned with 5 mL of methanol, 5 mL of Milli-Q water, 5 mL of HCl (pH 1) and finally 5 mL of Milli-Q water. Later, the eluted fraction was evaporated to dryness, recovered with 1 mL of methanol and filtered through a Durapore polyvinylidene fluoride filter (0.22 μm; Millipore Corp.) for HPLC-DAD and HPLC-qTOF MS analysis. Extraction were made in duplicate.

### 2.4. HPLC-DAD Analysis

To determine the intact glycosidic aroma precursors of grapes, an analysis was performed using an Agilent 1200 HPLC chromatograph (Agilent Technologies, Palo Alto, CA, USA) equipped with a DAD (Agilent G1315D; Agilent Technologies) coupled to an Agilent ChemStation (version B.03.01) data processing system. Taking as a reference the phenyl β-D-glucopyranoside spectrum DAD at 195 nm (Figure S1), the possible intact glycoside compounds were selected.

Separation was performed using an ACE C18-PFP column (4.6 mm × 150 mm, 3 μm particle size) connected to an Excel HPLC Pre-Column Filter 1PK (0.5 μm particle size) at 30 °C. Brisa LC2 C18 column (250 mm × 4.6 mm, 5 μm particle size) purchased from Teknokroma (Barcelona, Spain) was previously tested, but a worse results were obtained

The eluents used in both systems were water (A) and acetonitrile (B) with different gradients and different elution times. The flow rate was 0.8 mL/min, and the sample injection volume was 20 µL. The DAD (Agilent Technologies Deutschland GmbH, Wald bronn, Germany) was set at 190, 195, 210, 230, 280 and 324 nm. Each extract was injected in duplicate, so a total of 4 chromatograms were obtained.

### 2.5. HPLC-qTOF-MS Analysis

Identification of glycosidic aroma precursors previously selected using HPLC-DAD was performed using HPLC-qTOF MS, MassHunter PCDL Manager (Agilent Technologies) personal database and MS/MS fragmentation. For this purpose, the ACE C18-PFP column was used and the same solvents, flow rates and elution gradients to those used for the HPLC-DAD analysis were used to minimise any changes in the compounds’ retention time and the possible formation of adducts. Only the times of the peaks observed in HPLC-DAD chromatogram were tested in the HPLC-qTOF-MS and not all the peaks observed in this chromatogram. 20 μL of the sample were injected.

Samples were run in the negative mode using an Agilent 1290 Series II HPLC (Agilent Technologies Deutschland GmbH, Waldbronn, Germany) device coupled to an Agilent 6550 Q-TOF(Agilent Technologies Deutschland GmbH, Waldbronn, Germany) with a Jet Stream dual electrospray ionisation source. Nitrogen was used for both the drying gas and the sheath gas in the source. The capillary voltage was set to 4000 V, the nozzle voltage was set to 500 V and the fragmentor voltage was set to 350 V. The drying gas flow was set to 16 L/min at 150 °C, the sheath gas flow was set to 12 L/min at 300 °C and the nebuliser was set to 30 psig. The scan range was set to *m*/*z* 50–1100 for the MS and MS/MS mode. Before running each sequence, the instrument was calibrated according to the manufacturer’s specifications.

Reference mass ions were run continuously into the dual electrospray ionisation source at a rate of 50 μL/min throughout the run to ensure accurate mass calibration. The reference masses were 121.985587 and 1033.988109 *m*/*z*.

In the first run, MS/MS spectra were acquired using the target MS/MS mode of the instrument. The source conditions were the same as for single MS mode acquisition. The compounds were fragmented with a collision energy of 30 eV, and the precursor ions, established previously according to the literature, were acquired in a narrow isolation width (~1.3 u) to ensure ion selectivity with a minimum threshold of 200 counts. However, better results were obtained when the MS/MS spectra were acquired using the all ions MS/MS analysis mode of the instrument. The source conditions were again the same as for single MS mode acquisition, and three different collision energies (0, 10 and 40 eV) were selected for each run cycle.

## 3. Results

### 3.1. Isolation of Intact Glycosidic Aroma Precursor

The content of aroma glycosides in grapes is mainly concentrated in the skin, with a lower concentration in the pulp and juice [1]. Therefore, to isolate intact glycosides, grape skins were extracted using a microwave.

Glycosidic aroma precursors were extracted and purified using SPE with two different cartridges. The interference caused by free glucose and volatiles present in grapes needs to be removed. This was achieved through extraction using a LiChrolut EN cartridge (40–120 µm, 500 mg; Millipore Corp.). This is commonly used to analyse aroma precursors in grapes [23,24,25] since it resin was proved to be the most suitable for extracting precursor compounds and aromatic components [26] and it is particularly suitable for the extraction of polar analytes and a wide range of less polar solutions, such as hydroalcoholic solutions or wine [27]. Following the above procedure, an Oasis MCX SPE cartridge (60 µm, 500 mg; Waters Corp.) was used, which contains a mixture of reversed-phase and cationic-exchanger materials that allow the isolation of grape and wine flavonols, anthocyanin and other phenolic compounds [28,29,30]. Notably, these compounds coexist in grapes with aroma precursors and may lead to interference during chromatographic analyses. When the skin extract was passed through both cartridges, a decrease in phenolic compounds was observed, and hence a better glycosidic aroma precursor peak resolution was obtained.

### 3.2. HPLC-DAD Conditions

To choose the best HPLC-DAD conditions, two columns with different elution types were tested. First, separation was performed on a reversed-phase C_18_ column (Brisa LC2) because of its similarity to those used by some authors to analyse glycosidic phenolic compounds [31]. However, a better peak resolution was obtained with the ACE Excel 3 C18-PFP column, possibly because of its smaller particle size. In terms of elution, different gradients were tested with water (A) and acetonitrile (B) as eluents. The best results were observed with the following gradient for Solvent B: 0 min, 1%; 3 min, 2.5%; 13 min, 2.5%; 14 min, 5%; 34 min, 5%; 35 min, 9%; 35.5 min, 9%; 36 min, 10%; 56 min, 10%; 57 min, 11%; 57.5 min, 11%; 58 min, 12%; 78 min, 12%; 79 min, 15%; 83 min, 15%; 88 min, 100%; 93 min 100%. The wavelength used was 195 nm because this is the most adequate for our objective.

It should be noted that the literature has established a cut-off UV wavelength of acetonitrile of 190 nm [32], below which absorption may cause fluctuating baselines and higher noise levels. However, the measured UV absorbance may be influenced by several factors, such as the solvent’s purity, instrumental parameters or the reference substance [33]. Therefore, a single blank was run with the previously described gradient conditions and no interference on the baseline was observed.

### 3.3. Identification of Intact Glycosidic Aroma Precursors by HPLC-qTOF-MS

In general, in grapes, the monosaccharides glycosides are less present in grapes than disaccharides. In this line, Hjelmeland et al. 2015 [2] studied the monoterpene glycosides profile over three developmental stages in Muscat of Alexandria grapes, being monoterpenol hexose pentose the most abundant group. At time, Ghaste et al. 2015 [17] identified 15 glycosylated precursors of volatiles, mainly disaccharides glycosides. More reciently, Godshaw et al. 2019 [18] studied the intact profile of monoterpenyl glycosides in six *Vitis vinifera* grapes and they identified the malonylated monoterpenol glucosides, monoterpenol hexose-pentoses and monoterpendiol hexose-pentoses as predominated in all samples. In the same line, Caffrey et al. 2020 [34] described a method to characterize glycosidically bound precursors of monoterpenoids, norisoprenoids, volatile phenols, aliphatic alcohols and sesquiterpenoids in grapes. Therefore, before the analysis, a potential list of compounds was created on the basis of previously identified glycosidic aroma precursors in Muscat of Alexandria grapes [2,17,18,34].

All compounds were grouped according to their structure and molecular formula. A personal compound database was automatically generated by MassHunter PCDL Manager (Agilent Technologies, Deutschland GmbH, Waldbronn, Germany) using the exact mass of each compound according to the molecular formula imported. Final tentative identification was performed by comparing the fragmentation spectra with known glycoconjugate fragmentation patterns of these authors. These are the compounds included:

- *ALCOHOLS: Group C4: pentose-hexose* (C_15_H_28_O_10_; m.w. 368.1682); *pentose-hexose-hydroxy* (C_15_H_28_O_11_; m.w. 384.1632). *Group C5: pentose-hexose* (C_16_H_30_O_10_; m.w. 382.1839); *deoxyhexose-hexose* (C_17_H_32_O_10_; m.w. 396.1995); *pentose-hexose-hydroxy* (C_16_H_30_O_11_; m.w. 398.1788); *Group C6: pentose-hexose* (C_17_H_32_O_10_; m.w. 396.1995); *deoxyhexose-hexose* (C_18_H_34_O_10_; m.w. 410.2152); *pentose-hexose-dehydro-hydroxy* (C_17_H_32_O_11_; m.w. 412.1945); *hexose-hexose-dehydro* (C_18_H_32_O_11_; m.w. 424.1945); *hexose-hexose-hexose* (C_24_H_42_O_16_; m.w. 586.2473); *Group C8: pentose-hexose-dehydro-hydroxy* (C_19_H_36_O_11_; m.w. 440.2258).
- MONOTERPENOLS: *glycosides* (C_16_H_28_O_6;_ m.w. 316.1886); *hexose-pentose-glycosides* (C_21_H_36_O_10_; m.w. 448.2309); *malonylated glycosides* (C_21_H_34_O_11_; m.w. 462.2101); *hexose-deoxyhexose-glycoside* (C_22_H_38_O_10_; m.w. 462.2465); *dihexose-pentose-glycosides* (C_27_H_46_O_15_; m.w. 610.2837); *hexose-dipentose* (C_26_H_44_O_14_; m.w. 580.2731); *dipentose-hexose-glycosides* (C_26_H_44_O_14_; m.w. 580.2731).
- MONOTERPENEDIOLS: *hexose-pentose-glycoside* (C_21_H_36_O_11_; m.w. 464.2258); *dihydro-hexose-pentose-glycoside* (C_21_H_38_O_11_; m.w. 466.2414); *dihydro-deoxyhexose-hexose-glycoside* (C_22_H_38_O_11_; m.w. 478.2414); *pentose-hexose glycoside* (C_21_H_36_O_12_; m.w. 480.2207).
- MONOTERPENETRIOLS: *hexose-dehydro* (C_16_H_26_O_8_; m.w. 346.2414): *dihydro-hexose* (C_16_H_30_O_8_; m.w. 350.1941); *dihydro-hexose-pentose* (C_21_H_38_O_12_; m.w. 482.2362); *deoxyhexose-hexose* (C_22_H_38_O_12_; m.w. 494.2363).
- NORISOPRENOIDS: In this group of compounds, the aglycone were groups according to the database used by (Caffrey et al., 2020). *Norisoprenoids (Group G): hexose* (C_19_H_34_O_8_; m.w. 390.2254); *hexose-hydroxy* (C_19_H_34_O_9_; m.w. 406.2203); *hexose-hexose-hydroxy* (C_25_H_44_O_14_; m.w. 568.2731); *hexose-hexose* (C_25_H_44_O_13_; m.w. 552.2782). *Norisoprenoids (Group F): hexose* (C_19_H_39_O_8_; m.w. 388.2097); *hexose-hydroxy* (C_19_H_34_O_9_; m.w. 406.2203); *hexose-hexose* (C_25_H_42_O_13_; m.w. 550.2625). *Norisoprenoids (Group A): hexose-hexose* (C_25_H_42_O_13_; m.w. 550.2625). *Norisoprenoids (Group E): hexose* (C_19_H_30_O_8_; m.w. 386.1941); *hexose-pentose* (C_24_H_38_O_12_; m.w. 518.2363).
- *PHENOLS: pentose-hexose* (C_18_H_26_O_10_; m.w. 402.1500); *pentose-pentose-hexose* (C_25_H_40_O_13_; m.w. 548.2469).
- *SESQUITERPENOLS: hexose-pentose* (C_26_H_44_O_10_; m.w. 516.2934).

**Figure 2:**
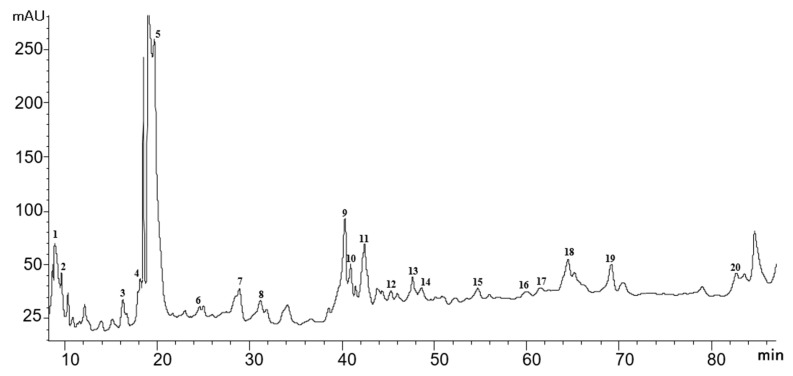
High-performance liquid chromatography with a diode array detector (HPLC-DAD) chromatogram at 195 nm with peaks tentatively selected as intact glycosidic aroma precursors in Muscat of Alexandria grapes.

As seen in Figure 2, 20 intact glycosidic aroma precursors were previously selected by using the HPLC-DAD method, and it was possible to identify aglycones in 12 of them by HPLC-qTOF-MS. All peaks selected as glycosidic aroma precursor were identified taken as a reference the phenyl β-D-glucopyranoside spectrum at 195 nm (Figure S1), which showed maximum UV absorbance at 195, 210 and 266 nm. This compound (C_12_H_16_O_6_, MW 256.25 g/mol) is commonly used as a standard in the analysis of glycosidic aroma precursors [7]. In all cases, the results indicated that there was a significant amount of absorption in the UV area. Specifically, the maximum absorption ranged from 190 to 230 nm, which could be attributed with sugars of the molecule by due to the similarity with their UV spectrum (Figure S2). Moreover, a shoulder was observed around 200–360 nm, which could be attributed to the aglycone.

**Figure 3:**
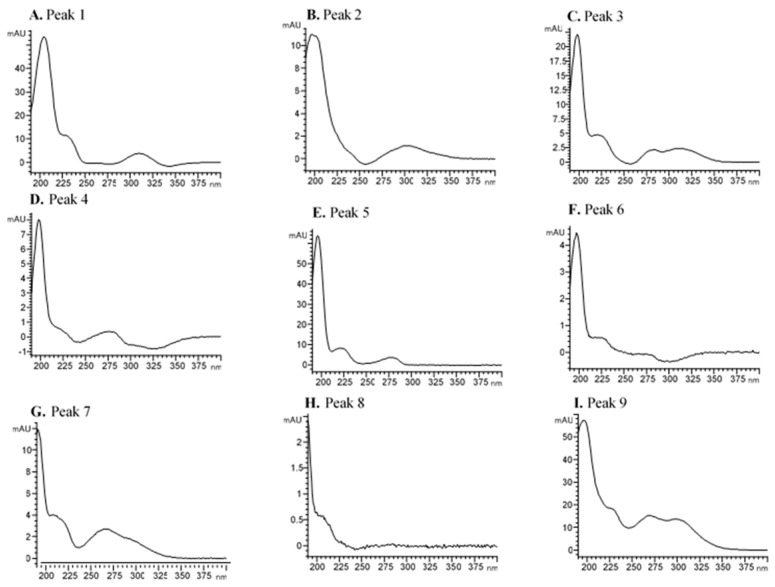

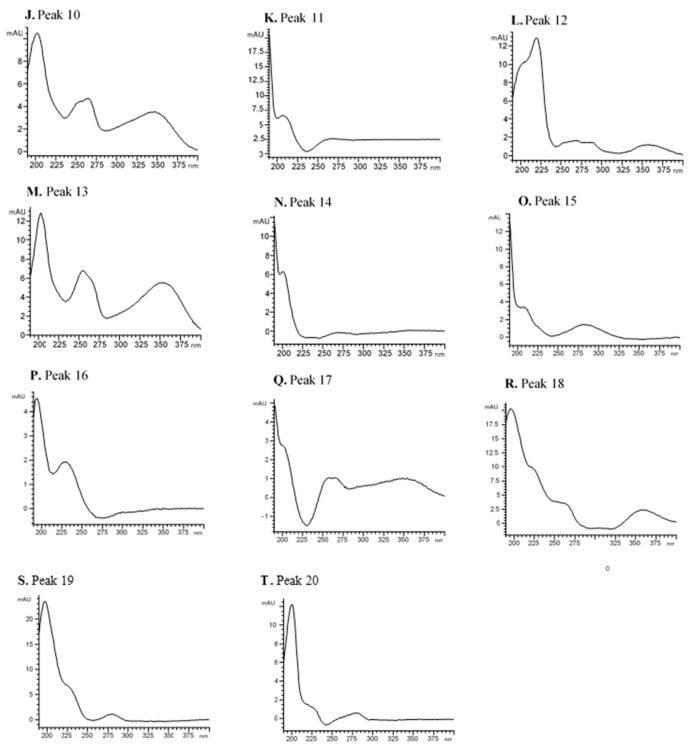
Intact glycosylated aroma precursor spectra (195 nm) of Muscat of Alexandria (peaks A-T). (**A**) Peak 1: C4 alcohol, RT 8.90. (**B**) Peak 2: Undefined glycoside 1, RT 9.74. (**C**) Peak 3: Undefined glycoside 2, RT 16.33. (**D**) Peak 4: C6 alcohol, RT 17.96. (**E**) Peak 5: Undefined glycoside 3, RT 19.68. (**F**) Peak 6: C6 alcohol, RT 24.49. (**G**) Peak 7: Undefined glycoside 4, RT 29.07. (**H**) Peak 8: Undefined glycoside 5, RT 29.07. (**I**) Peak 9: Dihydromonoterpentriol. RT 40.41. (**J**) Peak 10: Norisoprenoid. RT 41.43. (**K**) Peak 11: Undefined glycoside 6, RT 42.51. (**L**) Peak 12: Undefined glycoside 7, RT 45.29. (**M**) Peak 13: Monoterpendiol, RT 47.71. (**N**) Peak 14: Monoterpenetriol, RT 48.64. (**O**) Peak 15: Monoterpendiol, RT 54.89. (**P**) Peak 16: Monoterpendiol, RT 59.97. (**Q**) Peak 17: Monoterpendiol, RT 61.55. (**R**) Peak 18: Monoterpendiol, RT 64.47. (**S**) Peak 19: Monoterpendiol, RT 69.21. (**T**) Peak 20: Undefined glycoside, RT 82.69.

As seen in Figure 3, at 195 nm, it is possible to obtain a chromatogram in which glycosides are tentatively selected, when compared to the reference standard, but not all responses were similar, depending on the molar absorptivity at each specific wavelength. However, in the absence of a standard to perform correct quantification, the response can be approximated with the standard used.

To the identification of these peaks with the glycosylated precursors, MS/MS fragmentation at three different collision energies (10 and 40 eV) obtained by HPLC-qTOF MS/MS was studied (Table 1), taking as a reference the retention time of the HPLC-DAD chromatogram (±0.5 min). Peaks were confirmed as glycosidic aroma precursors by means of MS/MS fragmentation of sugar rings or sequential loss of sugars or aglycones from glycosides, and when possible aglycones were identified. The existence of sugars was confirmed by the presence of most or all of the following masses (*m*/*z*), associated with sugar ring fragments [2,18,34]: 59.0139, 71.0139, 73.0295, 85.0295, 89.0244, 101.0244, 113.0244, 115.0401, 119.0350, 125.0244 and 143.0350. Notably, according to Caffrey et al. (2020) [34], the specific sugar fragment *m*/*z* values included 131.0350 and 149.0455 for pentose sugars, 161.0455 and 179.0561 for hexose sugars and 145.0506 and 163.0612 for deoxyhexose sugars.

#### 3.3.1. Monoterpenediol Glycosides

Monoterpenediols are the polyhydroxylated form of monoterpenes (C_10_H_18_O, MW 170.2487 g/mol). In the studied grapes, six monoterpenediol hexose-pentose glycosides (MW 464.2258 g/mol) were selected (peak numbers 13, 15, 16, 18 and 19; Table 1). These compounds were recognised and confirmed using MS/MS analysis in the presence of [M-H]^−^ ions with *m*/*z* 463.2175. The first identification was performed in the presence of ions *m*/*z* 331.1755 and 293.0875 in the MS/MS spectra, corresponding to the neutral loss of a terminal pentose and aglycone unit, according to Caffrey et al. (2020) [34] and Godshaw et al. (2019) [18]. Moreover, the presence of both sugars was confirmed by their representative fragments: *m*/*z* 131.0344 and 149.0450 for the pentose sugar and *m*/*z* 161.045 and 179.0556 for the hexose sugar.

All compounds of this group appeared between 47 and 69 min on the chromatogram (Figure 2), at which point the elution gradient for acetonitrile ranged from 10% to 12%. The most abundant monoterpenediol glycoside appeared at 69.21 min (peak 19; Figure 2), with a DAD spectrum maximum at 197, 228 and 280 nm (Figure 3S). The second important monoterpenediol appeared at 64.47 min (peak 18; Figure 2), with shoulder wavelength absorbance at 196, 223, 262 and 358 nm (Figure 3R). Peak 15 (54.89 min; Figure 2) was the next abundant monoterpenediol, with a higher absorbance at 190, 205 and 282 nm (Figure 3O). Several researchers have pointed out that the most abundant monoterpenediols in Muscat of Alexandria grapes are 8-hydroxylinalool, 2,6-dimethyl-3,7-octadiene-2,6-diol, 3,7-dimethyl-1,5-octadiene-3,7-diol, 3,7-dimethyl-1,7-octadiene-3,6-diol, 8-hydroxynerol and 8-hydroxygeraniol [16,35,36,37]. Therefore, it could be associated with aglycone of the more important monoterpenediols identified above. Although these compounds make no direct contribution to aroma, they may break down to yield volatile compounds with a pleasant aroma, such as hotrienol and linalool, whose precursors are 3,7-dimethyl-1,5-octadiene-3,7-diol glycoside and 8-hydroxylinalool, respectively [37]. These compounds, along with other monoterpenes, such as geraniol, have been described as two of the most important compounds in wine, which contribute to the varietal characteristics of wine thanks to their flowery and sweet aroma nature [36,38].

#### 3.3.2. Monoterpenetriol Glycosides

One possible dihydromonoterpenetriol hexose pentose (MW 482.2363 g/mol) was selected in the studied grapes (peak 9; Table 1). Putative identification was performed in the presence of pentose (*m*/*z* 131.0344 and 249.0450) and hexose (*m*/*z* 161.045 and 179.0556) moieties and [M-H]^−^ ions with *m*/*z* 463.2175. Although no aglycone fragments were found in the fragmentation spectra, with no discernible fragments that can unequivocally identify this glycosidic compound, this is the only compound in our database with this parent ion. In the HPLC-DAD chromatogram, however, it was identified at 40.41 min (Figure 2), with 10% acetonitrile in the gradient. Notably, the spectrum of this compound showed three maxima at 197, 269 and 298 nm and a shoulder at 228 nm (Figure 3I). According to Caffrey et al. (2020) [34], the dihydromonoterpenetriol aglycone of this compound may be dihydroxy-dihydrolinalool (triol).

One monoterpenetriol hexose dehydro (MW 346.1628 g/mol) peak was also found (peak 14; Table 1). This compound was tentatively identified by the presence of an [M-H]^−^ ion parent with *m*/*z* 345.1628 and MS/MS product ion 183.1020, in line with the fragmentation proposed by Caffrey et al. (2020) [34]. The retention time in HPLC-DAD was 48.64 min (Figure 2), with an elution gradient similar to what has previously been used (i.e., monoterpenetriol). However, in this case, the DAD spectrum only showed two maximum absorbance values at 190 and 200 nm (Figure 3N).

#### 3.3.3. Norisoprenoid Glycosides

Norisoprenoids are a group of aroma compounds that originate from the oxidative degradation of carotenoids and contribute to the varietal characteristics of many types of wine, especially to aromatic varieties. In this study, peak 10 on the chromatogram (Figure 2) was correlated to a norisoprenoid (Group E) hexose pentose (MW 518.2363 g/mol) [34]. Similar to previously described glycosidic aroma precursors, identification was performed using a parent ion with *m*/*z* 517.2363 and product ions of MS/MS fragmentation *m*/*z* 205.1228, *m*/*z* 223.1332 and *m*/*z* 385.187, consistent with the fragmentation of Caffrey et al. (2020) [34]. Moreover, the presence of both sugars was confirmed by their representative fragments: *m*/*z* 149.0450 for the pentose sugar and *m*/*z* 161.045 and 179.0556 for the hexose sugar.

The retention time in the HPLC-DAD chromatogram was 41.43 min (Figure 2), with an elution gradient between 9% and 10% for acetonitrile. The DAD spectrum showed higher absorbance at 202, 257, 264 and 345 nm (Figure 3J). The aglycone of the detected norisoprenoid belonged to Group E of the above-mentioned glycosides, with 3,4-dihydroxy-β-ionone and 3-hydroxy-5,6-epoxy-β-ionone being the most abundant in the variety under study, which are associated with a pleasant flowery and fruity aroma.

#### 3.3.4. Alcohol Glycosides

In total, three intact aliphatic alcohol glycosides were tentatively identified in this study. Notably, peak 1 on the chromatogram (Figure 2) was identified as a C_4_ alcohol pentose hexose hydroxy (MW 384.1632 g/mol) by the presence of [M-H]^−^ ions with *m*/*z* 383.1550 and MS/MS product ions *m*/*z* 251.1136. This was the first glycosidic aroma precursor observed, which appeared with an elution gradient between 1% and 2.5% for acetonitrile, suggesting that it is the most polar compound among all the identified compounds. The absorbance peak of the spectrum was observed at 205 and 312 nm, as well as a shoulder at 231 nm (Figure 3A). The possible aglycone associated with it must have a molecular mass of 74.12 g/mol based on the number of carbons, it could be a C_4_ alcohol such as isobutyl alcohol.

Peaks 4 and 6 were associated with C_6_ alcohols hexose hexose dehydro (MW 424.1945 g/mol) by the presence of [M-H]^−^ ions with *m*/*z* 423.1945 and neutral loss of sugar or aglycone with *m*/*z* 261.1335. The first peak showed maximum absorbance at 198 and 276 nm and a shoulder at 221 nm (Figure 3D), whereas the second one showed a higher spectrum at 197 and 224 nm (Figure 3F).

Aliphatic alcohols are commonly associated with negative characteristics in grapes and wine, such as vegetable or herbaceous aroma [39]. However, when these compounds are present in a glycosidic form, they have a lower impact on the aroma of grapes and wine.

#### 3.3.5. Other Unconfirmed Peaks

In this study, DAD spectra corresponding to peaks 2, 3, 5, 7, 8, 11, 12 and 20 (Figure 2) as well as the subsequent association with MS/MS spectra were putatively identified as glycosidic conjugates, peak 5 being the most abundant of all those detected. Nevertheless, their MS/MS fragmentation was not associated with any of the groups of compounds described previously. Peaks 2, 3 and 5 were associated with monoglucosides as only characteristic fragmentation ions *m*/*z* 161.045 and 179.0556 for hexose were found. On the other hand, peaks 7, 8, 11 and 12 on the HPLC-DAD chromatogram were associated with disaccharides as, along with glucose ion fragmentation, ions *m*/*z* 131.0344 and 149.0450 for pentose were detected. Finally, peak 20 on the chromatogram was associated with a trisaccharide, pentose hexose deoxyhexose glycoside, because the fragmentation pattern was found to share the ion *m*/*z* 163.0613 that is associated with deoxyhexose.

The fact that there aren’t commercial standard of glycosidic aroma precursors of grapes makes it difficult their quantification. However, the use of an internal standard such as, for example, the phenyl β-D-glucopyranoside used as a reference in this article, it could allow to make an approximate quantification of them.

## 4. Conclusions

In this study, an innovative method for analysing intact glycosidic aroma precursors in grapes was developed. In total, 20 glycosidic aroma precursors were selected using HPLC-DAD and tentatively identified by HPLC-qTOF-MS/MS. Moreover, all interferences due to free sugars and polyphenols were resolved using LiChrolut EN and Oasis MCX cartridges, respectively. It was also found that disaccharide glycosides, mainly monoterpenediols and alcohols, are the most abundant form in Muscat of Alexandria grapes.

The results obtained in this study prove that it is possible to determine intact glycosidic aroma precursors in grapes by a widely used analytical technique as HPLC-DAD, making it useful in viticulture or oenology to study the impact of vineyard treatments on the aroma potential of grapes and wines.

## Figures and Tables

**Table 1:** Intact glycosidic aroma precursors tentatively identified by HPLC-qTOF-MS/MS and the correspondent area peaks by HPLC-DAD in Muscat of Alexandria grapes.

*Peak Number*	*RT*	*Compound Name*			*ESI MS/MS*				*DAD*	
		Sugars	Aglycone	Formula	Exact Mass	*m/z* [M-H]^-^	*m/z* Experimental [M-H]^-^	MS/MS Products Ions	λ Max (UV)	Peak Area (λ195)
1	8.90	Pentose Hexose Hydroxy	C4 alcohol	C_15_H_28_O_11_	384.1632	383.1554	383.1559	59.0135; 71.0131; 73.0288; 89.0234; 101.0236; 113.0236; 119.0492; 149.0082; 161.0439; 179.054; 251.1123; 383.1552	205; 231; 312	753 ± 41
2	9.74	Hexose	Undefined aglycone 1	NF	NF	NF	NF	59.0135; 71.0132; 73.0288; 89.0235; 101.0237; 113.0237; 161.0439; 163.0392; 179.0536	196; 297	240 ± 15
3	16.33	Hexose	Undefined aglycone 2	NF	NF	NF	NF	59.0139; 71.0135; 73.0292; 89.0238; 101.0241; 113.024; 119.0343; 161.0444; 366.1191; 443.193; 444.1957	198; 218; 284; 313	575 ± 49
4	17.96	Hexose Hexose Dehydro	C6 alcohol	C_18_H_32_O_11_	424.1945	423.1867	423.1872	59.0139; 71.0136; 73.0292; 89.0239; 101.0241; 113.024; 119.0348; 125.024; 161.0445; 179.0551; 243.1229; 261.1337; 423.1856	198; 221; 276	406 ± 42
5	19.68	Hexose	Undefined aglycone 3	NF	NF	NF	NF	59.014; 71.0137; 73.0294; 89.0239; 101.0242; 119.0347; 161.0446; 179.0552; 287.1496; 403.1604	196;220;276	8415 ± 545
6	24.49	Hexose Hexose Dehydro	C6 alcohol	C_18_H_32_O_11_	424.1945	423.1867	423.1872	59.0139; 71.0137; 73.0294; 89.024; 101.0242; 113.0242; 119.0346; 161.0446; 179.0556; 261.1335; 423.1868	197; 224	229 ± 22
7	29.07	Pentose Hexose	Undefined aglycone 4	NF	NF	NF	NF	59.0141; 71.0139; 89.0241; 101.0244; 119.0347; 131.0347; 149.045; 161.0449; 179.0557; 347.1169; 359.1346; 429.2129	191; 265	845 ± 73
8	33.58	Pentose Hexose	Undefined aglycone 5	NF	NF	NF	NF	59.0142; 71.0139; 73.0295; 89.0242; 101.0245; 113.0245; 119.035; 131.035; 149.0451; 161.045; 179.0559; 193.0501; 379.1607	190; 207	265 ± 26
9	40.41	Pentose Hexose	Dihydromonoterpentriol	C_21_H_38_O_12_	482.2363	481.2285	481.2291	59.0142; 71.0139; 89.0243; 101.0245; 113.0245; 119.0348; 131.0348; 143.0346; 149.0451; 161.0449; 179.0556; 481.2283;	197; 228; 269; 298	651 ± 45
10	41.43	Pentose Hexose	Norisoprenoid	C_24_H_38_O_12_	518.2363	517.2285	517.2291	59.0143; 71.0140; 73.0296; 89.0243; 101.0245; 113.0245; 119.0348; 125.0244; 143.0345; 149.0453; 161.0449; 179.0554; 205.1228; 223.1332; 385.1870; 517.2283	202; 257; 264; 345	111.93 ± 8
11	42.51	Pentose Hexose	Undefined aglycone 6	NF	NF	NF	NF	59.0142; 71,014; 73,0295; 89,0243; 101,0245; 113,0245; 119,0349; 143,0345; 149,0448; 161,0449; 287,1495; 449,2027	190; 202	961 ± 56
12	45.29	Pentose Hexose	Undefined aglycone 7	NF	NF	NF	NF	59,0143; 71,014; 73,0296; 89,0243; 101,0246; 113,0245; 119,035; 149,0452; 161,045; 179,0558; 287,1499; 449,2031	220; 267; 359	195 ± 15
13	47.71	Pentose Hexose	Monoterpendiol	C_21_H_36_O_11_	464.2258	463.218	463.2175	59.0143; 71.0140; 89.0243; 101.0246; 113.0245; 119.0349; 131.0351; 149.0451; 161.0450; 179.0557; 463.2175	203; 254; 352	339 ± 36
14	48.64	Hexose Dehydro	Monoterpene triol	C_16_H_26_O_8_	346.1628	345.155	345.1555	59.0143; 71.0140; 73.0296; 89.0243; 101.0246; 113.0245; 119.0348; 131.0345; 143.0346; 149.0449; 161.0449; 179.0555; 183.1020; 345.1559	190; 200	267 ± 22
15	54.89	Pentose Hexose	Monoterpendiol	C_21_H_36_O_11_	464.2258	463.2258	463.2185	59.0143; 71.014; 89.0243; 101.0246; 113.0246; 119.0351; 131.0348; 149.0451; 161.045; 179.0558; 293.0875 *; 331.1755 *; 463.2185	190; 205; 282	387 ± 29
16	59.97	Pentose Hexose	Monoterpendiol	C_21_H_36_O_11_	464.2258	463.218	463.2185	59.0143; 71.0141; 89.0244; 101.0247; 113.0247; 119.0351; 125.0246; 131.0353; 149.0453; 161.045; 179.0557; 293.0877 *; 331.1756 *; 463.2185	193; 231	239 ± 19
17	61.55	Pentose Hexose	Monoterpendiol	C_21_H_36_O_11_	464.2258	463.218	463.2186	59.0144; 71.0141; 89.0244; 101.0247; 113.0247; 119.0351; 131.035; 143.0348; 149.0453; 161.045; 163.0613; 179.0558; 293.0875 *; 331.1754 *; 463.2185	190; 204	208 ± 21
18	64.47	Pentose Hexose	Monoterpendiol	C_21_H_36_O_11_	464.2258	463.218	463.2185	59.0143; 71.0141; 73.0297; 89.0244; 101.0246; 113.0249; 119.0352; 131.0347; 143.0347; 149.0451; 161.0450; 179.0556; 331.1754 *; 463.2185	196; 223; 262; 358	763 ± 59
19	69.21	Pentose Hexose	Monoterpendiol	C_21_H_36_O_11_	NF	NF	463.2185	59.0144; 89.0244; 101.0248; 119.0357; 161.0449; 179.0555; 331.1755 *; 463.219	197; 228; 280	848 ± 89
20	82.69	Pentose Hexose Deoxyhexose	Undefined aglycone 8	NF	NF	NF	NF	59.0144; 73.0297; 89.0244; 101.0248; 119.0353; 149.0452; 161.0451; 163.0613; 179.0557; 301.1653	200; 226; 279	618 ± 62

* MS/MS sugar of aglycone loss; RT: Retention time (min); NF: nof found; peak area: mean value (*n* = 4) ± standard deviation.

## Data Availability

The data presented in this study are available on request from the corresponding author.

## Supplementary Materials

The following are available online at https://www.mdpi.com/2304-8158/10/1/191/s1, Figure S1: Diode array detector (DAD) spectra of phenyl β-D-glucopyranoside (195 nm), Figure S2: Diode array detector (DAD) spectra of D-glucose (maximum at 192 nm).
