# Supplementary material for: Analysis of Intact Glycosidic Aroma Precursors in Grapes by High-Performance Liquid Chromatography with a Diode Array Detector
Source: Foods. 2021 Jan 19;10(1):191. doi: 10.3390/foods10010191 (PMC7832828; doi:10.3390/foods10010191)
Supplement: Supplementary file 1 [file foods-10-00191-s001.pdf]

Supplementary Materials:

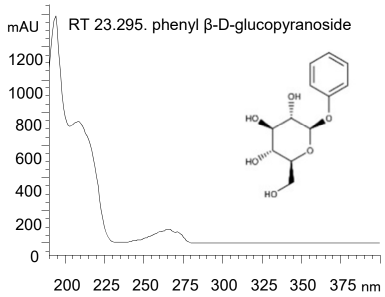

Figure S1. Diode array detector (DAD) spectra of phenyl β-D-glucopyranoside (195 nm).

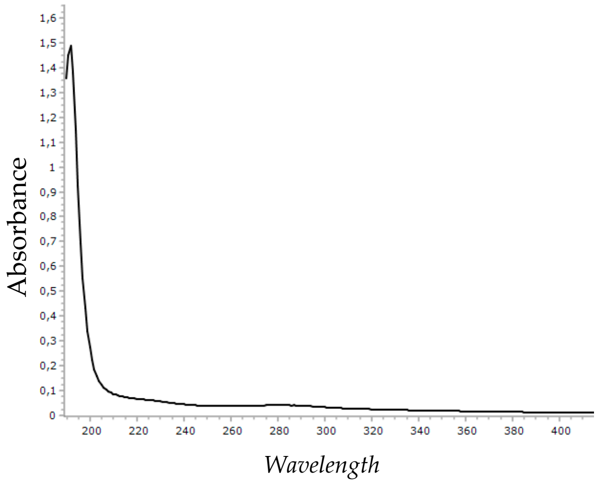

Figure S2. Diode array detector (DAD) spectra of *D*-glucose (maximum at 192 nm).
